# Preparation of Complex Polysaccharide Gels with *Zanthoxylum bungeanum* Essential Oil and Their Application in Fish Preservation

**DOI:** 10.3390/gels10080533

**Published:** 2024-08-13

**Authors:** Shan Xue, Chao Li, Zhouyi Xiong

**Affiliations:** 1 College of Biological Science and Technology, Minnan Normal University, Zhangzhou 363000, China; lichao22@mnnu.edu.cn; 2 Research Institute of Zhangzhou-Taiwan Leisure Food and Tea Beverage, Zhangzhou 363000, China; 3 Zhangzhou Food Science Research Institute, Zhangzhou 363000, China; 4 School of Life and Health Technology, Dongguan University of Technology, Dongguan 523808, China; xiongzhouyi@dgut.edu.cn

**Keywords:** *Zanthoxylum bungeanum* essential oil (ZEO)-complex polysaccharide gels, preparation, MATLAB analysis, box-Behnken design, fish preservation

## Abstract

In this study, novel functional ZEO-complex gels were prepared using sodium alginate, inulin, grape seed extract (GSE), and *Zanthoxylum bungeanum* essential oil (ZEO) as the primary raw materials. The effect of the addition of inulin, GSE, and ZEO on water vapor permeability (WVP), tensile strength (TS), and elongation at break (EAB) of ZEO-complex polysaccharide gels was investigated. A comprehensive score (Y) for evaluating the characteristics of ZEO-complex polysaccharide gels was established by principal component analysis. MATLAB analysis and box-Behnken design describe each factor’s four-dimensional and three-dimensional interactions. It was found that Y could reach the maximum value when the ZEO addition was at a moderate level (C = 2%). The optimum preparation process of ZEO-complex polysaccharide gels was as follows: the addition of inulin was at 0.84%, the addition of GSE was at 0.04%, and the addition of ZEO was at 2.0785%; in this way, the Y of ZEO-complex polysaccharide gels reached the maximum (0.82276). Optical scanning and X-ray diffraction tests confirmed that the prepared ZEO-complex gels have a smooth and continuous microstructure, good water insulation, and mechanical properties. The storage test results show that ZEO-complex polysaccharide gels could play a significant role in the storage and fresh-keeping of grass carp, and the physicochemical properties of complex polysaccharide gels were improved by adding ZEO. In addition, according to the correlation of fish index changes during storage, adding ZEO in complex polysaccharide gels was closely correlated with the changes in fish TBARS and TVB-N oxidation decay indices. In conclusion, the ZEO-complex polysaccharide gels prepared in this study had excellent water insulation, mechanical properties, and outstanding fresh-keeping effects on grass carp.

## 1. Introduction

In recent years, renewable packaging materials have become increasingly popular. Finding natural active ingredients from natural macromolecular materials to prepare biodegradable and even edible gel products can not only solve the problem of environmental pollution, but also promote the innovation and development of food processing and storage, such as plastic packaging, and has become the focus of current scholars’ research [1]. Natural macromolecular polysaccharide is a kind of renewable energy and is characterized by biocompatibility, among others. Natural polysaccharide gels can be applied to biomedicine and food packaging materials [2]. However, the gels made of a single substance have relatively poor mechanical properties and higher water vapor transmission rate, which is insufficient to meet the actual needs. Therefore, it is necessary to improve the physical and chemical properties of polysaccharide gels by adding functional substances to extend the shelf life of food and effectively keep perishable food fresh [3,4].

Inulin is a reserved polysaccharide in plants. It is a linear polysaccharide formed from D-fructose linked by a β (1→2) glucoside bond. It has significant antioxidant [5], anti-inflammatory, antiviral, and biological functions, such as regulating intestinal flora, blood sugar, and blood lipids, and is widely used in food and drug fields [6,7]. Applying inulin in the preparation of complex polysaccharide gels can help improve the gels’ biological efficacy, but relevant research is still rare.

Sodium alginate (SA), a natural polymer derived from brown algae, contains many hydroxyl and carboxylic groups and can be used as a food stabilizer, binder, and thickener [8]. The gels prepared by SA can prevent food quality loss, texture, and color change, maintaining flavor and delaying fat oxidation [9]. However, the composite polysaccharide gels with SA as the main component have some problems, such as solid moisture permeability and poor water resistance, so it is necessary to combine them with some other elements. Studies have shown that, in recent years, research on composite polysaccharide gels used in food packaging, preservation, and other fields has gradually been based on edible substances [10]. Adding natural active ingredients such as plant essential oils [11] and anthocyanin [12] can effectively improve the gels’ mechanical, antioxidant, and antibacterial properties.

*Zanthoxylum bungeanum* essential oil (ZEO) is extracted from the pericarps of *Zanthoxylum bungeanum*. It is mainly composed of Linalool, D-limonene, β-laurene, α-pinene, and other active ingredients [13], and provides good inhibition against a variety of foodborne bacteria [14]. Chen et al. [13] proved that ozone-coated chitosan/konjac glucomannan loaded with ZEO can be successfully applied to food packaging, providing a new idea for the research and development of active packaging materials. On the one hand, the addition of ZEO can improve the biological activity of polysaccharide gels; on the other hand, it can enhance the physical and chemical properties of the gels, such as increasing the hydrophobicity of polysaccharide gels and improving their mechanical properties, which has great application potential [15,16].

Grape seed extract (GSE) is a class of polyphenols extracted and isolated from grape seeds, which has suitable antibacterial and antioxidant activities [17]. GSE is an active botanically derived product consisting of flavonoids, such as naringenin, ascorbic acid, tocopherols, and citric acid [18]. Due to the characteristics of stable source, mature production process, high safety, and water solubility, GSE has shown good application in meat preservation research. Research showed that grape seed extract has been applied to Chitosan/corn starch-based active packaging film [19], pullulan polysaccharide/xanthan gum apple film [20], regenerated cellulose film [21], and other polysaccharide gels.

This study took ZEO, inulin, SA, and grape seed extract as the primary raw materials, supplemented by ice acetic acid and glycerin. It optimized the preparation process through single factor variable analysis (the addition of inulin, the addition of GSE, and the addition of ZEO), principal component analysis, and the response surface optimization method so that a new kind of ZEO-complex polysaccharide gels was developed. In addition, the ZEO-complex polysaccharide gels were applied to store and preserve fish in a thin film to provide a theoretical basis for the innovation and application of polysaccharide gel products.

## 2. Results and Discussion

### 2.1. Results of Single Factor

#### 2.1.1. Effect of the Addition of Inulin on Water Vapor Permeability (WVP), Tensile Strength (TS), and Elongation at Break (EAB) of the ZEO-Complex Polysaccharide Gels

**Figure 1:**
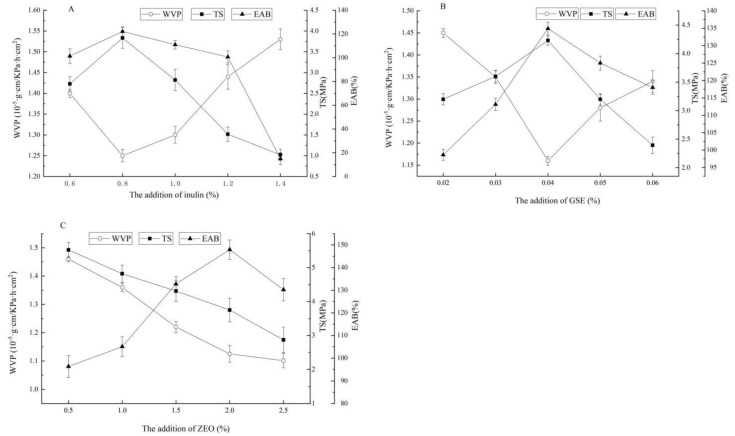
Effect of the addition of inulin, the addition of GSE, and the addition of ZEO on WVP, TS, and EAB of the ZEO-complex polysaccharide gels. (**A**) the addition of inulin; (**B**) the addition of GSE; (**C**) the addition of ZEO.

As shown in Figure 1A, with the increase in inulin, ZEO-complex polysaccharide gels showed a trend of WVP firstly decreasing and then significantly increasing (*p* < 0.05), and WVP had a minimum value when the addition of inulin was at 0.8%. Both TS and EAB showed a trend of increasing and then significantly decreasing (*p* < 0.05). When the addition of inulin was at 0.8%, both TS and EAB had large values. Therefore, the addition of inulin was determined at 0.8% for subsequent tests. Inulin had high hydrophilicity, and the addition of an appropriate amount of inulin can improve the stability of the gel, increasing the degree of cross-linking between molecular chains, but too much inulin may destroy the stable three-dimensional structure of the ZEO-complex gels system, enhance the water permeability, and affect the mechanical properties of polysaccharide gels [22].

#### 2.1.2. Effect of the Addition of GSE on WVP, TS, and EAB of the ZEO-Complex Polysaccharide Gels

As shown in Figure 1B, with the increase in GSE, the WVP of ZEO-complex polysaccharide gels first significantly decreased and then significantly increased (*p* < 0.05). When the addition of GSE was at 0.04%, the WVP value was the smallest. The TS and EAB of ZEO-complex polysaccharide gels increased continuously when the addition of GSE was at 0.02~0.04% and decreased significantly when the addition of GSE was at 0.04~0.06% (*p* < 0.05). Therefore, the addition of GSE was determined at 0.04% for the follow-up tests. GSE is rich in polyphenols, which can change the interaction between molecular chains in the polysaccharide membrane matrix and the flexibility of the film [23]. Similar results are observed in anthocyanins and other macromolecular polymers, such as the carboxymethyl cellulose/starch and purple potato side anthocyanin indicator film prepared by Jiang et al. [24] and the pH-sensitive smart film based on SA and purple sweet potato peel extract prepared by Zhao et al. [25].

#### 2.1.3. Effect of the Addition of ZEO on WVP, TS, and EAB of the ZEO-Complex Polysaccharide Gels

It can be seen from Figure 1C that, with the increase in ZEO, both the WVP and TS of ZEO-complex polysaccharide gels showed a gradual downward trend. There was no significant difference in the WVP of ZEO-complex polysaccharide gels when the dosage of ZEO was at 2.0% and 2.5% (*p* > 0.05). However, EAB showed a trend of significant increase at first and then a significant decrease (*p* < 0.05). When ZEO was added at 2%, the WVP of ZEO-complex polysaccharide gels had a more substantial value. Therefore, based on the changes in each index, the additional amount of ZEO was determined at 2% for the follow-up tests. The research of Chen et al. [13] showed that the addition of 1% of ZEO could improve the TS of the chitosan/OKGM film supported by the ZEO film, and reduce the water solubility, WVP, and water content of the polysaccharide gel films, but too much ZEO addition may reduce the mechanical property of the polysaccharide gels. The polysaccharide film loaded with plant essential oil has a broad market prospect in the field of food packaging, and it provides a new idea for the research and development of active packaging materials.

### 2.2. Results of MATLAB Analysis and Box-Behnken Design

#### 2.2.1. Results of MATLAB Analysis

MATLAB has a robust scientific calculation function, which can effectively calculate the optimal solution through programming code to establish mathematical models [26,27]. At present, MATLAB has been widely used in process optimization, and the reports in the field of food mainly come from the optimization of the edible oil extraction process [28], grape seed polyphenol extraction process [29], pineapple peel residue polyphenol extraction process [30], and so on.

**Figure 2:**
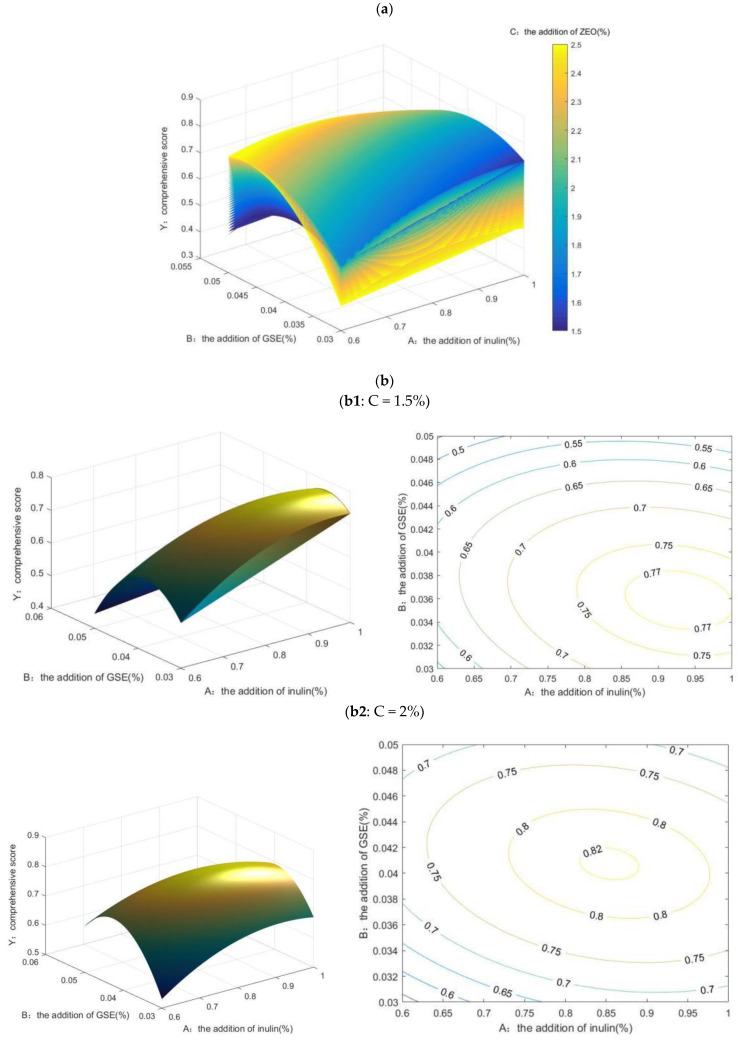

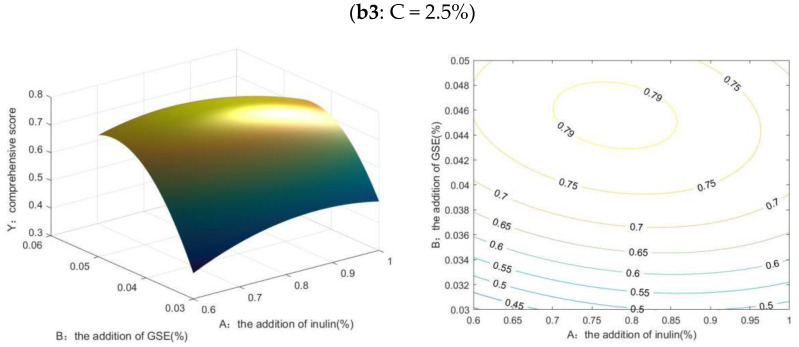
Four-dimensional (**a**) and three-dimensional (**b**) renderings of the effects of the addition of inulin (A), the addition of GSE (B), and the addition of ZEO (C) on the comprehensive score (Y).

Through modeling, four-dimensional and three-dimensional renderings of the effects of the addition of inulin (A), the addition of GSE (B), and the addition of ZEO (C) on the comprehensive score (Y) were obtained (Figure 2a,b).

To better describe the interactive effects among the analysis data, three-dimensional rotary surface and contour projection maps of the interactive effects of the addition of inulin (A) and the addition of GSE (B) on the comprehensive score (Y) were drawn, respectively, when the ZEO supplemental level (C) was low (1.5%), medium (2%), and high (2.5%) (Figure 2b1,b2,b3).

When the ZEO supplemental level was set as low (C = 1.5%), the B value was fixed, and the Y value gradually increased with the increase in A. At this time, the value of Y ranged from 0.4444 to 0.7777. Y obtained a more significant value when A was 0.85~1% and B was 0.035~0.038% (Figure 2b1). When the ZEO supplemental level was set as moderate (C = 2%), the B value was fixed, and with the increase in A the Y value first increased and then decreased. In this case, the value of Y ranged from 0.5313 to 0.8219. When A was 0.80~1%, and B was 0.04~0.042%, Y obtained a more significant value (Figure 2b2). When the ZEO supplemental level was set as high (C = 2%), the A value was fixed, and the Y value gradually increased and then decreased with the increase in B. In this case, the value of Y ranged from 0.3932 to 0.7989. Y obtained a more significant value when A was 0.7~0.85% and B was 0.044~0.048% (Figure 2b3).

In summary, Y can reach the maximum value when ZEO addition is at a moderate level (C = 2%).

#### 2.2.2. Results of Box-Behnken Design

**Figure 3:**
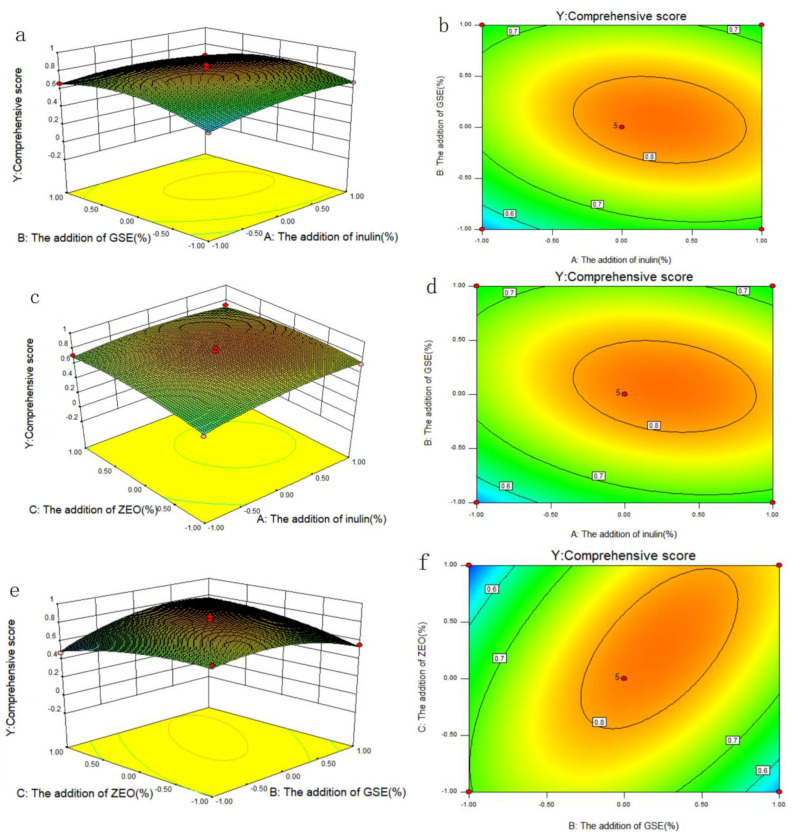
Response surface plots (**a**,**c**,**e**) and contour plots (**b**,**d**,**f**) of the effects of the interaction of various factors on comprehensive score.

Based on the results of the single factor analysis, the addition of inulin (A), GSE (B), and ZEO (C) were selected as the investigation factors. The experimental design and results are shown in Table 1. The response surface plots (a,c,e) and contour plots (b,d,f) of the effects of the interaction of each factor on the comprehensive score (Y) are shown in Figure 3. The coefficients of the models were calculated, and the predicted model was as Formula (1):

Y = 0.82 + 0.035A + 0.028B + 0.009825C − 0.035AB − 0.028AC + 0.11BC − 0.060A^2^ − 0.13B^2^ − 0.065C^2^ (1)

The results of ANOVA for the quadratic model are shown in Table 1 (Y), indicating that the model was highly appropriate for the prediction. The F-value and *p*-value determined the significance of each coefficient. Generally, the larger the F value is, the smaller the *p* value is. Thus, it can be concluded from the F value of the Y model that the model was significant (*p* < 0.05).

Moreover, the higher the coefficient of determination (R^2^) (0.9535), the better the reasonability of the model. Overall, the fraction of the variation in the response by the model (R^2^), adjusted R^2^ and regression *p*-value, and lack of fit values > 0.05 all indicated that the model fitted the experimental data points well [31]. The adequate precision for the system was 11.683 (>4). Therefore, a sufficient signal was obtained [32]. The response surface and contour maps of the pairwise interactions of factors A and B, A and C, B and C are shown in Figure 3a,b, Figure 3c,d and Figure 3e,f, respectively.

As can be seen from Figure 3a,c, the slope of the response surface was relatively gentle, indicating that the interaction between AB and AC was not significant (*p* > 0.05). In contrast, the slope of the response surface in Figure 3e was steeper. The contours were distributed in a uniform and tight micro-oval shape, indicating that the interaction between BC was significant. The results of ANOVA are consistent with the result seen in Table 1 (P_AB_ (0.093 > 0.05), P_AC_ (0.1669 > 0.05), and P_BC_ (0.0005 < 0.05)). The interaction between factors was detected in all the samples, mainly in the order BC (the addition of GSE and ZEO) > AB (the addition of inulin and GSE) > AC (the addition of inulin and ZEO).

### 2.3. Results of Verify Test

**Figure 4:**
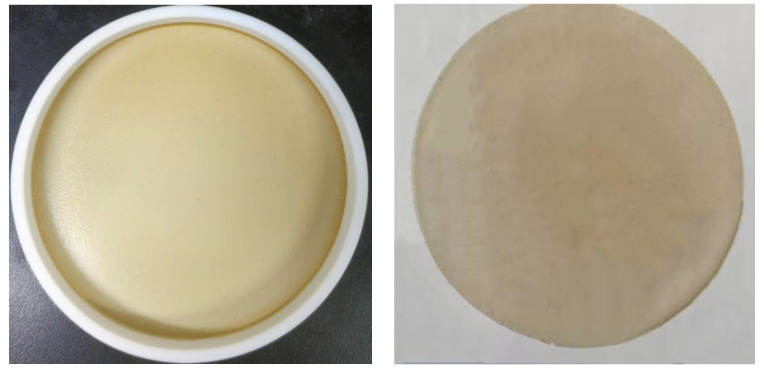
The samples of prepared ZEO-complex polysaccharide gels. (The picture on the **left** showed the gel on a tetrafluoroethylene plate, and the picture on the **right** showed the gel on white paper.)

Based on the model analysis, the optimal experimental conditions were as follows: the addition of inulin was at 0.8418%, the addition of GSE was at 0.0415%, and the addition of ZEO was at 2.0785%. The predicted Y of the ZEO-complex polysaccharide gels characteristics was 0.82276. The actual Y value (0.8215 ± 0.011) was close to the expected value (*p* > 0.05). Therefore, the experiments showed that the model was reliable. The results of MATLAB and box-Behnken design are highly consistent. The obtained ZEO-complex polysaccharide gels are shown in Figure 4.

### 2.4. Results of X-ray Diffraction (XRD)

**Figure 5:**
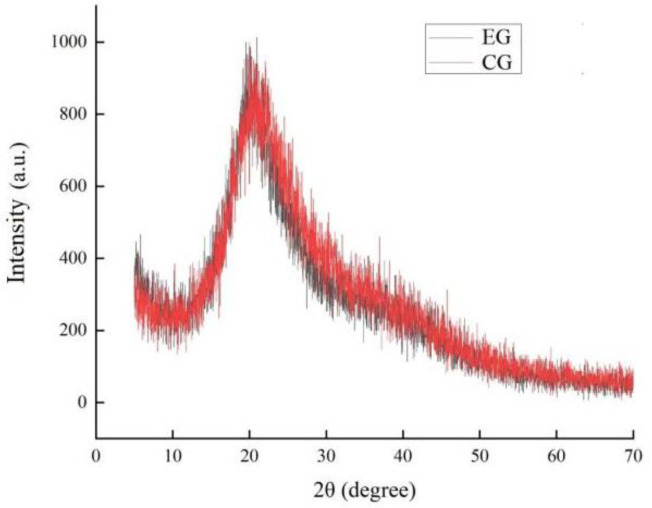
XRD of the experimental group (EG) (with ZEO added) and the control group (CG) (without ZEO).

Crystallinity is closely related to the properties and structure of composite films and is an important parameter to characterize the materials in composite films. The size of crystallinity is closely associated with the material’s stability and the molecules’ arrangement in the gels. With the increase in crystallinity, the melting point of the molecules also increases. Crystallization is a molecular arrangement which is proportional to the molecular melting point. The crystalline region is regularly arranged, and the non-crystalline region is randomly placed. Substances with high crystallinity show narrow and high peaks; low crystallinity materials exhibit peak width and low peak shape [33]. It can be seen from Figure 5 that the complex polysaccharide gels of the experimental group (EG) (with ZEO added) and the control group (CG) (without ZEO) had a sharp high absorption peak at about 2θ = 20.7°, indicating that the crystallinity corresponding to their positions was high. The complex polysaccharide gels and ZEO formed by SA, inulin, and grape seed extract all have amorphous properties. After the combination of the complex polysaccharide gels and ZEO, no other diffraction peaks appear, indicating that ZEO can be compatible with the complex polysaccharide gels, and the peak shape was narrow and sharp, indicating that the ZEO-composite polysaccharide gels had high crystallinity and good thermal stability, which was consistent with the views proposed by Augustine et al. [33].

### 2.5. Analysis of Scanning Electron Microscopy

**Figure 6:**
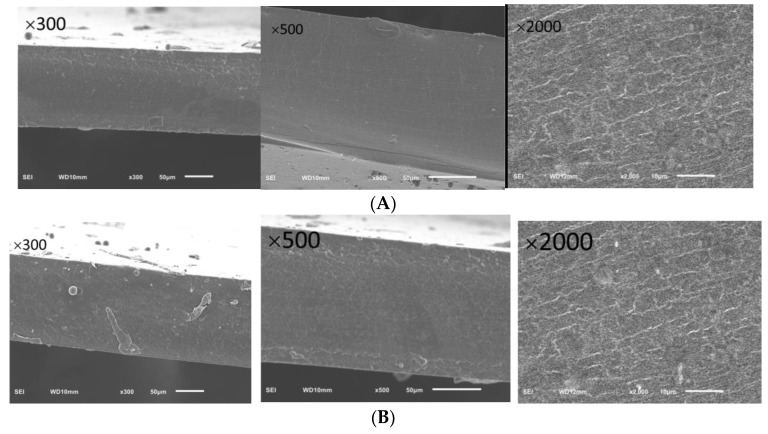
Cross-section and surface microstructure of EG and CG. The grass carp preserved with ZEO-complex polysaccharide gels was the experimental group (EG), and the grass carp preserved with complex polysaccharide gels without ZEO was the control group (CG). (**A**) Cross-section and surface microstructure of EG; (**B**) cross-section and surface microstructure of CG.

The microstructure diagram of the EG cross-section and surface is shown in Figure 6A, and the microstructure diagram of the CG cross-section and surface is shown in Figure 6B. The 300× and 500× magnifications were cross-section images, and the 2000× magnifications were surface images. As can be seen from Figure 6, the surface and cross-section of the complex polysaccharide gel (EG) supplemented with ZEO were much smoother than those without ZEO (CG). This may be because ZEO, due to its smaller particle size, infiltrates into different polymers of the complex polysaccharide gels, thereby reducing the presence of bubbles and porosity and improving the performance of SA/inulin/grape seed extract complex polysaccharide gels to a certain extent [23], which also validated the optimization effect based on the barrier properties and mechanical properties described above. The reason for this phenomenon may be that GSE contains multiple ortho-hydroxyl groups, which can provide strong hydrogen supply capacity, and can be tightly cross-linked and evenly distributed in the membrane matrix through various forces such as hydrogen bonding, hydrophobic action, and covalent cross-linking, thus improving the order of the membrane structure [23]. In addition, Guo et al. [34] showed that adding a certain number of natural extracts could improve the roughness of the membrane surface, which may be because the addition of natural extracts fills the pores between molecules and makes the molecular connections more orderly.

### 2.6. Physical and Chemical Quality Changes in Grass Carp during Storage

#### 2.6.1. Change in Water Holding Capacity

**Figure 7:**
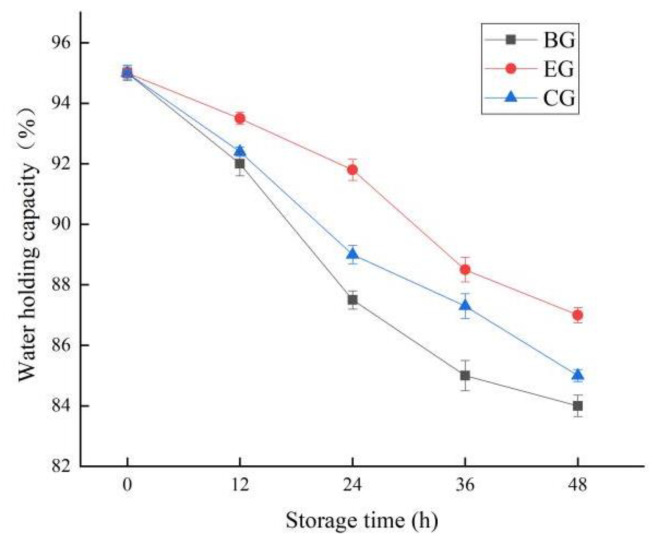
Changes in water holding capacity of fish during storage. The grass carp preserved with ZEO-complex polysaccharide gels was the experimental group (EG), the grass carp preserved with complex polysaccharide gels without ZEO was the control group (CG), and the grass carp preserved with nothing was the blank group (BG)).

Water retention is an important factor affecting fish tenderness. As can be seen from Figure 7, within 48 h of storage at 25℃, with the extension of storage time, the water holding capacity of the experimental group (EG) supplemented with ZEO, the control group (CG) without ZEO, and the blank group (BG) without any preservation measures showed a significant decreasing trend (*p* < 0.05). It was not difficult to see that the water holding capacity of CG was significantly higher than that of BG, and the water holding capacity of EG supplemented with ZEO was better than that of CG, which was because the addition of ZEO can improve the water insulation of polysaccharide gels. This was consistent with the previous research results. In addition, due to its good film forming properties and water retention [35], the complex polysaccharide can effectively reduce the water loss of fish.

#### 2.6.2. Change in pH Value

**Figure 8:**
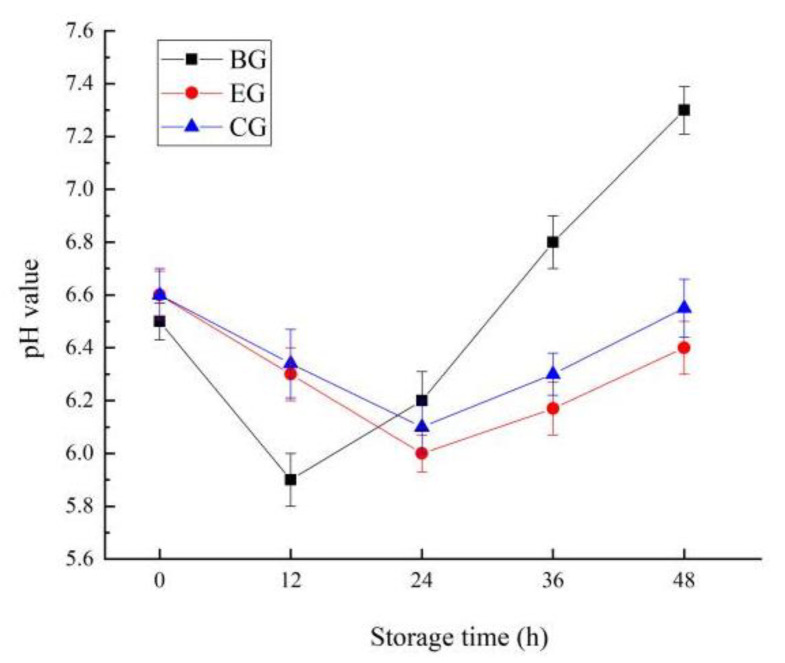
Changes in pH value for fish during storage. The grass carp preserved with ZEO-complex polysaccharide gels was the experimental group (EG), the grass carp preserved with complex polysaccharide gels without ZEO was the control group (CG), and the grass carp preserved with nothing was the blank group (BG).

Changes in pH affect the color, water retention, flavor, and shelf life of meat. During storage at 25 °C, the pH value of the fish was changed, as shown in Figure 8. The changes in EG and CG were similar. During 1~24 h storage, the pH values of the two groups gradually decreased; after 24~48 h storage, the pH values of the two groups continued to rise. At the later stage of storage, the pH value of CG increased faster than that of EG. For BG, the pH value of fish decreased significantly during 0~12 h. It increased considerably during 12–48 h storage (*p* < 0.05), and the increasing range was substantially more significant than that of EG and CG. This may be because the strong barrier of EG can slow the oxidative corruption of fish, inhibit the generation of amines and ammonia to a certain extent, and weaken the rise in pH value during storage [36].

#### 2.6.3. Change in TVB-N

**Figure 9:**
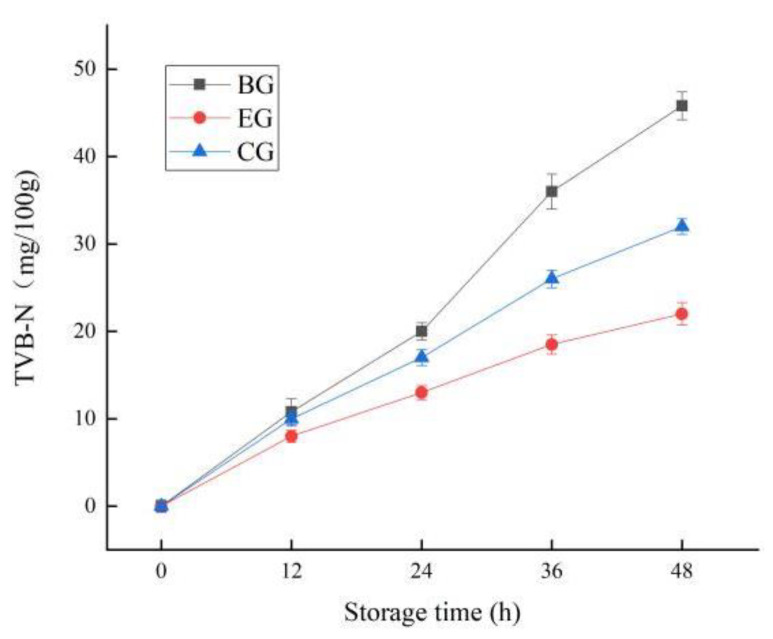
Changes in TVB-N for fish during storage. The grass carp preserved with ZEO-complex polysaccharide gels was the experimental group (EG), the grass carp preserved with complex polysaccharide gels without ZEO was the control group (CG), and the grass carp preserved with nothing was the blank group (BG).

TVB-N is an important index to evaluate the freshness of fish. With the extension of storage time, the protein in fish meat will be decomposed into amino acids and some small molecules under the action of bacteria, and then cause the aggregation of volatile base compounds, which is one of the critical factors affecting the odor of meat [37]. As seen in Figure 9, the TVB-N content in the EG, CG, and BG increased continuously during storage, and the rate of increase was BG > CG > EG. The slow rise in TVB-N in the EG was closely related to the antibacterial activity of ZEO and the barrier property of ZEO-complex polysaccharide gels. In recent years, the application of plant essential oils and polysaccharides in the preservation of fish and meat has been widely investigated. On the one hand, their complex can act as an antibacterial agent, acting on the cell membrane and cytoplasm of microorganisms to prevent the degradation of protein; on the other hand, the complex can act as a natural preservative, effectively resisting the oxidative rancidity of lipids [38]. This is consistent with the results of this paper.

#### 2.6.4. Change in TBARS

**Figure 10:**
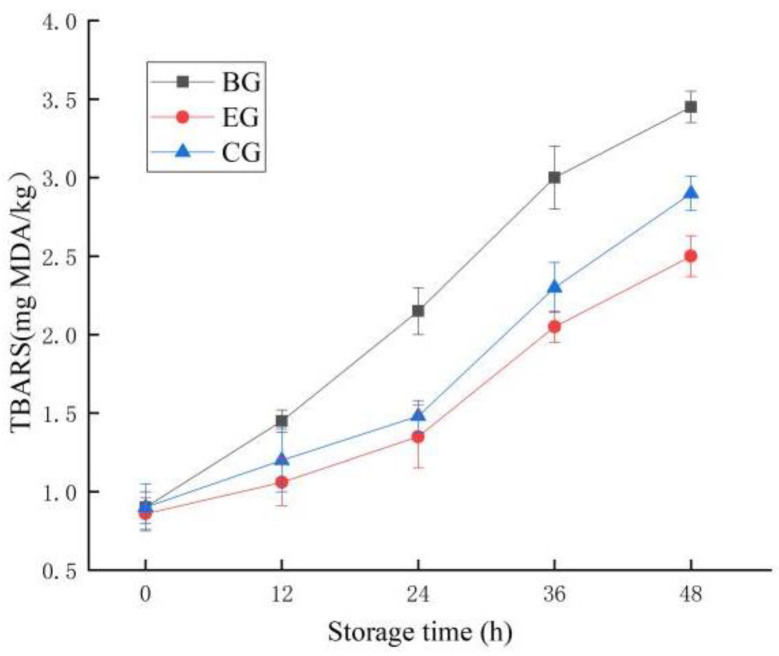
Changes in TBARS for fish during storage. The grass carp preserved with ZEO-complex polysaccharide gels was the experimental group (EG), the grass carp preserved with complex polysaccharide gels without ZEO was the control group (CG), and the grass carp preserved with nothing was the blank group (BG).

TBARSs are secondary oxidation products of lipids, such as aldehydes and ketones, which are the cause of undesirable rancid odor in food. The higher the TBARS value, the higher the degree of fat oxidation and the greater the degree of meat spoilage [39]. Similarly, as can be seen from Figure 10, the TBARS values of the EG, CG, and BG were significantly increased (*p* < 0.05). Among them, the increase in the BG was the fastest, and that in the EG was slower. Similarly, the antibacterial properties of ZEO and the blocking properties of ZEO-complex polysaccharide gels can significantly inhibit the oxidation of lipids and the formation of bacteria in fish meat so that it can inhibit the increase in TBARS value during fish meat storage more effectively than in the BG and CG. This may be because the plant essential oil and polysaccharide compound can form a special nano emulsion, and this nano emulsified essential oil can be a good substitute for plant essential oil, which can be directly applicated in meat preservation. The mechanism of action may be realized through hydrogen atom transfer, electron transfer, and the ability to chelate transition metals [40]. This is also a question worthy of further exploration in this study.

#### 2.6.5. Change in Sensory Score

**Figure 11:**
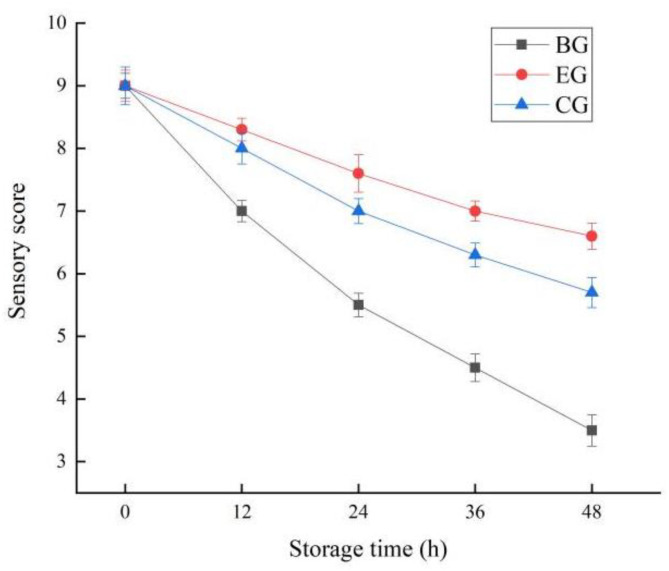
Changes in sensory score of fish during storage. The grass carp preserved with ZEO-complex polysaccharide gels was the experimental group (EG), the grass carp preserved with complex polysaccharide gels without ZEO was the control group (CG), and the grass carp preserved with nothing was the blank group (BG).

The changes in the sensory scores of fish during storage are shown in Figure 11. It can be seen from the figure that the sensory scores of the EG, CG, and BG all showed a downward trend, with the most significant decline in the BG and the smallest decline in the EG. In terms of color, the luster of the fillets in the three groups decreased gradually, and the yellowness of the fish in the BG was more prominent. Regarding odor, fish coated with gels (EG and CG) had less fishy taste during storage, while BG had more fishy taste and EG had less fishy taste and had a light ZEO aroma. In terms of morphology, there was no mucous on the surface of the fish from the EG and CG, while the mucous was more evident in the BG at 36 h. In summary, the combination of plant essential oils (such as ZEO) and active packaging materials to prepare active packaging can achieve a controlled delivery of essential oils to packaged foods, enhance the performance of the packaging system, and achieve the purpose of maintaining the sensory quality of food and extending the food’s shelf life [41]. The complex polysaccharide gel film containing plant essential oil has good biocompatibility and antibacterial activity. At the same time, plant polysaccharides are also a natural active ingredient, and their combination not only makes the film have excellent barrier property, but also effectively protects the sensory properties of perishable food by virtue of its antibacterial activity, and extends the shelf life of food [42].

#### 2.6.6. Correlation Analysis of the Change in Each Index

**Figure 12:**
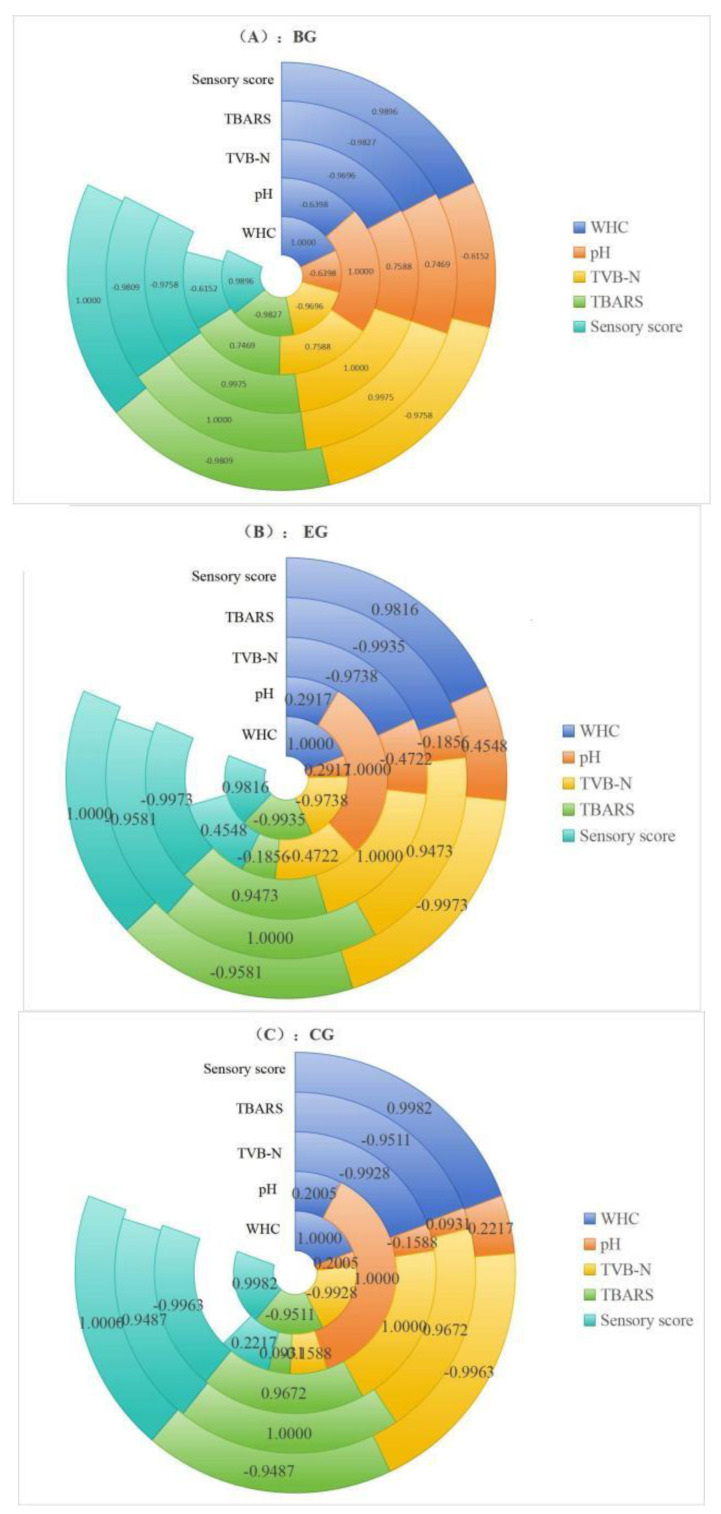
Correlation analysis of the change in each index. (**A**) BG; (**B**) EG; (**C**) CG. The grass carp preserved with ZEO-complex polysaccharide gels was the experimental group (EG), the grass carp preserved with complex polysaccharide gels without ZEO was the control group (CG), and the grass carp preserved with nothing was the blank group (BG).

The correlation of the fish indices of the BG, EG, and CG during storage is shown in the multilayer pie chart in Figure 12A–C, respectively, and the longer the ring, the stronger the correlation. The result of the correlation analysis shows that WHC was closely positively correlated with the changes in sensory scores, TVB-N, and TBARS, WHC was significantly negatively correlated with the changes in TVB-N and TBARS, and sensory scores were significantly negatively correlated with the changes in TVB-N and TBARS in the BG. The correlation analysis results of the EG, CG, and BG indicators are similar. Still, the difference was that, in the EG and CG, the correlation between pH value and other indicators was relatively weak. The correlation between sensory score and TVB-N changes was higher, while the correlation between sensory score and TVB-N changes was weaker. This indicated that the complex polysaccharide gels affected fish preservation to a certain extent, so the changing trend in pH value, TVB-N, and TBARS of fish in the CG and EG had more significant changes than in the BG.

Moreover, the correlation between WHC and TBARS in the EG was more significant than that in the CG, which also showed that WHC and TBARS in the EG had different trend changes from the CG during storage. These results indicate that adding ZEO had a noticeable effect on lipids. This may be because fat is easily oxidized under the action of trace water and oxygen, and the addition of ZEO can block water and oxygen from passing through the complex polysaccharide gels [13], which was consistent with the previous conclusion. In addition, the shelf life of fish preserved by ZEO-complex polysaccharide gels can be modeled and predicted in the later stage, based on the change in TBARS and TVB-N, and the molecular mechanism of ZEO-complex polysaccharide gels on preservation can be deeply discussed.

## 3. Conclusions

In this study, the preparation process of ZEO-complex polysaccharide gels was optimized by the single factor control variable method, principal component analysis, MATLAB four-dimensional and three-dimensional analysis, as well as box-Behnken design, and the gels were applied to the preservation of grass carp. When the addition of inulin was at 0.8418%, the addition of GSE was at 0.0415%, and the addition of ZEO was at 2.0785% (moderate addition), the comprehensive score of the ZEO-complex polysaccharide gels can obtain the maximum theoretical value (0.82276). The prepared ZEO-complex polysaccharide gels have good water insulation and mechanical properties. The storage test results show that ZEO-complex polysaccharide gels could play a significant role in the storage and fresh-keeping of grass carp, and that the addition of ZEO improves the physicochemical properties of complex polysaccharide gels. They can significantly delay the oxidative spoilage of fish fat and protein. The shelf life of ZEO-complex polysaccharide gels can be predicted based on the changes in TBARS and TVB-N, and the preservation mechanism can be further discussed. In addition, the follow-up research can focus on improving the comprehensive properties of ZEO-complex polysaccharide gels, especially the mechanical and biological properties of gels, and carry out technical research on pilot-scale, scale-up, and large-scale production. This may further improve the protective effect of ZEO-complex polysaccharide gels on the physicochemical indices and sensory quality of fish and other perishable foods.

## 4. Materials and Methods

### 4.1. Material

*Zanthoxylum bungeanum* essential oil (ZEO) was provided by Duomai (Fujian) Food Co., Ltd. (Fujian, China). The Zanthoxylum bungeanum essential oil was extracted by the method of supercritical CO_2_ extraction, and the extraction rate was about 13.05%. Inulin, SA (SA), and grape seed extract (GSE) (polyphenol content > 95%) were all food grade and purchased from Zhejiang Yinuo Biotechnology Co., Ltd. Zhejiang China. Glacial acetic acid, glycerin, 2-thiobarbituric acid, petroleum ether, benzene, chloroform, and methanol were analytically pure and brought from Xilong Scientific Co., Ltd. (Shantou, China). Water was purified with a Milli-Q water purification system (Millipore Co., Ltd., Burlington, MA, USA). Boron trifluoride methanol solution and 37 fatty acid methyl esters standard-mixed were brought from Shanghai Anpu Experimental Technology Co., Ltd. Fresh grass carp (about 1.5 kg weight) was purchased from RT-Mart (Zhangzhou, China) and transported to the laboratory alive.

### 4.2. Preparation of ZEO-Complex Polysaccharide Gels

Add 2 mL glacial acetic acid to 100 mL deionized water, and then add 1.2% SA and a certain amount of GSE (the final concentration was 0.02~0.06%). Stir them in a magnetic stirrer at 50 °C for 20 min until the above ingredients are completely dissolved, and then add a certain amount of inulin (the final concentration was 0.6~1.4%). The mixture must be stirred until completely dissolved. Next, add 1 mL of glycerol to the mixed solution, and add 0.5~2.5% ZEO. The above-mixed system is homogenized at high speed for 5 min at 10,000 r/min to obtain the solution of ZEO-complex polysaccharide. Using the method of casting coating, 25 mL of film liquid is uniformly poured into the polyethylene dish, then dried in the oven at 45 °C for 9 h to obtain the sample of ZEO-polysaccharide gels. The gels are removed and placed in a dry bottle with 25 ± 1 °C and relative humidity of 50 ± 5% for 24 h for water balance.

### 4.3. Single Factor Experiment

Investigate the effects of three single factors (the addition of inulin (0.6~1.4%), the addition of GSE (0.02~0.06%), and the addition of ZEO (0.5~2.5%)) on the water vapor permeability (WVP), elongation at break (EAB), and tensile strength (TS) of the film.

### 4.4. Characterization of ZEO-Complex Polysaccharide

#### 4.4.1. Thickness

The thickness of ZEO-complexly saccharide gels was tested with a digital micrometer (BMD-25D, Mitutoyo Mfg. Co., Ltd., Kawasaki, Japan). Five random regions were selected, and the average values were calculated.

#### 4.4.2. Water Vapor Permeability (WVP)

WVP was carried out according to the work of Fasihi et al. [43]. Briefly, add 20 g silica gel to the centrifuge tube to keep the RH value of the tube at 0%, and seal the centrifugal tube with thin ZEO-complex polysaccharide gels with a diameter slightly larger than the centrifugal tube. Place the centrifugal tube in an environment with a temperature of 25 °C and an RH value of 60%, and record the weight change in the weighing cup every two hours. The WVP values were calculated as Formula (2):

(2) $$WVP=\frac{1}{A}(\frac{∆W}{t})\times \frac{T}{P({RH}_{1}-{RH}_{2})}$$

where ΔW represents the weight change before and after measurement (g); t is the time (s); A is the test area (m^2^); P is the saturation vapor pressure of water (3.169 × 10^3^ Pa at 25 °C); RH_1_ denotes the relative humidity value in the desiccators; RH_2_ denotes the relative humidity value in the permeation cell; and T is the ZEO-complex polysaccharide gels thickness (mm).

#### 4.4.3. Mechanical Properties

The texture analyzer determined the TS and EAB (CT3-10K, American Bollerfly Company, Middleboro, MA, USA). The ZEO-complex polysaccharide gels were cut into 8.0 cm × 2.0 cm strips. The initial clamping distance was 60 mm, and the crosshead velocity was 50 mm/min [44].

#### 4.4.4. X-ray Diffraction (XRD) Analysis of ZEO-Complex Polysaccharide Gels

The ZEO-complex polysaccharide gels were analyzed using the X-ray diffraction technique. This was performed with an X-ray diffractometer (X’Pert Pro-A Analytical Raleigh, NC, USA) (PANalytical B.V., Alemlo, The Netherlands) that operates at 45 k voltage and 40 mA current. The pattern was recorded with Cu Kα radiation in a 2θ configuration [45].

#### 4.4.5. Scanning Electron Microscope (SEM)

Thin ZEO-complex polysaccharide gels were fractured in liquid nitrogen to prepare a cross-section. After gold spraying under vacuum, the surface and cross-section morphology of ZEO-complex polysaccharide gels were observed by SEM (SU-8000) (Hitachi, Ltd., Tokyo, Japan) at an accelerating voltage of 20 kV.

#### 4.4.6. Calculation of Comprehensive Score of ZEO-Complex Polysaccharide Gels

Referring to the method of Jiang et al. [46], the comprehensive score of ZEO-complex polysaccharide gels was indicated by the membership degree of the comprehensive score and the analysis of the principal component. The principal component analysis was mainly used to determine the weight of each index.

Membership degree was calculated according to Formulas (3) and (4):

(3) $$P=\frac{A_{i}-A_{min}}{A_{max}-A_{min}}$$

(4) $$P=1-\frac{A_{i}-A_{min}}{A_{max}-A_{min}}$$

where P is membership degree; A_i_ is the corresponding index value; A_max_ is the maximum value of the corresponding indicator; A_min_ is the minimum value of the corresponding indicator.

The positive effect (TS and EAB) values were calculated using Formula (3). The negative effect (WVP) values were calculated by Formula (4).

The comprehensive score of ZEO-complex polysaccharide gels (S) was calculated according to Formula (5):

(5) $$S=a_{1}P_{1}+a_{2}P_{2}+a_{3}P_{3}$$

In the Formula (5), P_1_ to P_3_ were the membership values of TS, EAB, and WVP, respectively; a_1_ to a_3_ were the weights of the three indicators.

### 4.5. Four and Three-Dimensional Analysis by MATLAB and Box-Behnken Design

The response values were the comprehensive score of the ZEO-complex polysaccharide gels (S), and the three variables were the addition of inulin (0.6~1.0%), the addition of GSE (0.03~0.05%), and the addition of ZEO (1.5~2.5%).

Using MATLAB software (R2021a), through the preparation of the program M (program code), the four-dimensional interaction effects of the addition of inulin (A), GSE (B) and ZEO (C) on the comprehensive score of ZEO-complex polysaccharide gels were calculated.

### 4.6. The Application of the ZEO-Complex Polysaccharide Gels on the Preservation of Grass Carp

#### 4.6.1. Storage Experiment

Each fresh grass carp (2.4 kg ice: 3.6 L water: 1.5 kg fish) was humanely slaughtered with ice-water slurry, and immediately beheaded, scaled, eviscerated, and washed with tap water. The fish was cut into 2 cm ×2 cm × 0.2 cm fillets. The methodological protocol of the current study was approved by the welfare and ethics committee in the animal experiments of the College of Biological Science and Technology, Minnan Normal University [47].

The samples were stored at 25 °C for 0, 12, 24, 36, and 48 h. The changes in water content, TVB-N value, pH value, TBARS value, fatty acid composition, and sensory score were determined at each period. The prepared ZEO-complex polysaccharide gels were coated on the fish. The grass carp preserved with ZEO-complex polysaccharide gels was the experimental group (EG), and the grass carp preserved with complex polysaccharide gels without ZEO was the control group (CG).

#### 4.6.2. Change in Water Holding Capacity (WHC)

Put 3.00 g of fish wrapped with filter paper into the centrifugal tube, record the initial weight of the fish as M_0_, centrifuge it at 4500 rpm for 5 min, wipe the water on the surface of the fish with the wrapped filter paper, and record the weight as M_1_. The WHC of the fish was calculated according to Formula (6).

(6) $$\mathrm{WHC}\;\left(\%\right)=\frac{M1}{M0}\times 100$$

#### 4.6.3. pH Value

The calibrated portable pH meter (Testo735-2, Testo AG, Neustadt, Germany) was used to test the pH value. Drill a hole into the fish, and insert the electrode of the pH meter. The electrode probe should be vertically inserted into the sample, avoiding flat or even inverted positions, to prevent bubbles inside the electrode, which would affect the determination result. Each sample was measured at three different locations, and the results are expressed as the average of the three trials [48].

#### 4.6.4. TVB-N Value

The content of TVB-N was determined by the Kjeldahl method (Kjeltec 8400, FOSS, Hiller) [49]. The fat of the fish sample was removed and the fish was stirred. A total of 10 g of sample was weighed in a conical flask, and 100 mL of distilled water was added. Then, the mixture was shaken and left to soak for 30 min before straining. The conical bottle containing the absorber and indicator solution was placed at the lower end of the condensing tube, and the lower end of the condensing tube was under the absorbed liquid level in the conical bottle. A certain amount of sample filtrate was precisely absorbed into the reaction chamber of the still, a certain amount of magnesium oxide was added to the suspension, and at the same time, the reaction chamber was rapidly plugged and water was added to prevent air leakage. The absorbent was titrated with a standard solution of hydrochloric or sulfuric acid, and the endpoint was blue-purple. The result is expressed as mg of nitrogen per 100 g of sample.

#### 4.6.5. Thiobarbituric Acid Reactive Substance (TBARS) Analysis

The content of TBARS was determined by the Xue method [48]. A total of 5 g of chopped fish was added into 25 mL 7.5% trichloroacetic acid solution. The mixed system was homogenized at 9000 r/min for 1 min, and rested at 4 °C for 1 h, and then centrifuged at 5000 r/min for 10 min. Then, 5 mL of supernatant was added to the 5 mL 0.02 moL/L TBA solution. The above system was heated in boiling water for 30 min and cooled for 1 h; then, 5 mL of chloroform was added, shaken well, and stratified. The supernatant was taken at the wavelength of 532 nm and 600 nm to determine the absorbance. The result is expressed as mg of malondialdehyde (MDA) per kg of sample (Formula (7)).

(7) $$TBARS\;(mg/1000)=\frac{A_{532nm}-A_{600nm}}{155}\times 72.06\times 100$$

#### 4.6.6. Sensory Evaluation

Ten food students (5 male and 5 female) who passed the training, were healthy, and had no bad habits were selected to form a sensory evaluation group to conduct the sensory evaluation of odor, color, tissue, and elasticity on the fish samples. Sensory scores were performed on the samples according to Table 2, and the results are expressed as averages.

### 4.7. Statistical Analysis

The collected data were subjected to ANOVA using Excel^®^ 2010 software and Tukey tests (with a 95% confidence interval) to evaluate differences between the results. MATLAB (MATrix LABoratory) software (R2021a) was used for interactive test data calculation and four-dimensional plotting. The principal component analysis used SPSS 17.0. The optimization experiment was conducted by using design-expert 8.0.6.

## Figures and Tables

**Table 1:** Experimental design plan, results, and variance analysis of response surface experiment. ** indicates that the difference is very significant, *p* < 0.01.

Test	The Addition of Inulin (A)	The addition of GSE (B)	The Addition of ZEO (C)	WVP (10^−5^·g·cm/KPa·h·cm^2^)	TS (MPa)	EAB (%)	Comprehensive Score (Y)
1	1 (1%)	0 (0.04%)	1 (2.5%)	1.39	3.00	68.75	0.7335
2	0 (0.8%)	0	0 (2%)	1.15	4.50	79.60	0.8642
3	1	0	−1 (1.5%)	1.43	2.73	68.62	0.7186
4	1	−1 (0.03%)	0	1.45	1.41	100.00	0.6657
5	0	0	0	1.21	2.86	39.06	0.7862
6	0	1 (0.05%)	−1	1.59	2.34	45.79	0.5526
7	−1 (0.6%)	1	0	1.47	2.63	66.24	0.6623
8	1	1	0	1.52	2.16	64.37	0.6613
9	−1	0	−1	1.54	1.35	59.78	0.5931
10	0	−1	−1	1.41	2.15	93.50	0.7254
11	−1	0	1	1.43	2.73	68.62	0.7186
12	0	0	0	1.16	4.12	77.25	0.8221
13	−1	−1	0	1.55	1.43	58.06	0.5273
14	0	1	1	1.38	3.15	68.23	0.7421
15	0	−1	1	1.60	1.54	73.52	0.4741
16	0	0	0	1.17	3.80	78.78	0.8145
17	0	0	0	1.18	3.50	81.55	0.7941
Source	Sum of squares	Mean square	F Value			*p* Value	
Model	0.18	0.02	15.95			0.0007	**
A	0.009647	0.009647	7.5			0.0289	
B	0.006373	0.006373	4.96			0.0613	
C	0.0007722	0.0007722	0.6			0.4637	
AB	0.004858	0.004858	3.78			0.093	
AC	0.003058	0.003058	2.38			0.1669	
BC	0.049	0.049	37.79			0.0005	**
A^2^	0.015	0.015	11.73			0.0111	**
B^2^	0.068	0.068	53.02			0.0002	**
C^2^	0.018	0.018	14.02			0.0072	**
Residual	0.008998	0.001285					
Lack of Fit	0.005268	0.001756	1.88			0.2735	not significant
Pure Error	0.00373	0.0009325					
Cor Total	0.19						

**Table 2:** Criteria for sensory evaluation.

Score	Odor	Color	Tissue	Elasticity
9–10	Fish smell was rich, no odor	White	Close section, clear texture	Rapid recovery after compression
7–8	Fish smell, no odor	Yellowish white	Partial close and clear	Slow recovery after pressing
5–6	Fish smell was light, slight odor	Yellowish	Not tight, but not loose	Recovery after compression is slow
3–4	Fishy and unpleasant smell	Grayish yellow	Soft section, partial loose	It is difficult to recover after pressing
1–2	Very fishy and smelly	Gray	Oar-shaped and loose	It will not recover after compression

## Data Availability

The original contributions presented in the study are included in the article, further inquiries can be directed to the corresponding author.
