# Correction: Xue et al. Preparation of Complex Polysaccharide Gels with *Zanthoxylum bungeanum* Essential Oil and Their Application in Fish Preservation. *Gels* 2024, *10*, 533

**DOI:** 10.3390/gels11120967

**Published:** 2025-12-01

**Authors:** Shan Xue, Chao Li, Zhouyi Xiong

**Affiliations:** 1 College of Biological Science and Technology, Minnan Normal University, Zhangzhou 363000, China; lichao22@mnnu.edu.cn; 2 Research Institute of Zhangzhou-Taiwan Leisure Food and Tea Beverage, Zhangzhou 363000, China; 3 Zhangzhou Food Science Research Institute, Zhangzhou 363000, China; 4 School of Life and Health Technology, Dongguan University of Technology, Dongguan 523808, China; xiongzhouyi@dgut.edu.cn

The authors would like to make the following correction to [1]: In the original publication, the insertion of Figure 3d was incorrect. It was repeated with Figure 3b. Figure 3d has now been inserted correctly as follows:

In Figure 6A, the third figure is a mistake. The corrected Figure 6 appears below.

The authors state that the scientific conclusions are unaffected. This correction was approved by the Academic Editor. The original publication has also been updated.

## Figures and Tables

**Figure 3:**
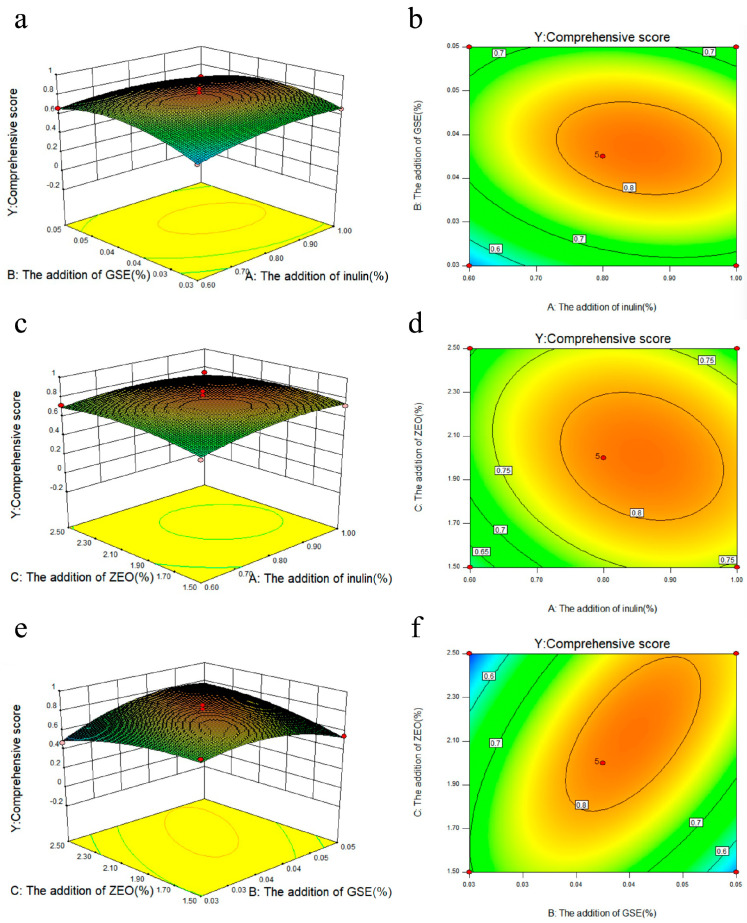
Response surface plots (**a**,**c**,**e**) and contour plots (**b**,**d**,**f**) of the effects of the interaction of various factors on comprehensive score.

**Figure 6:**
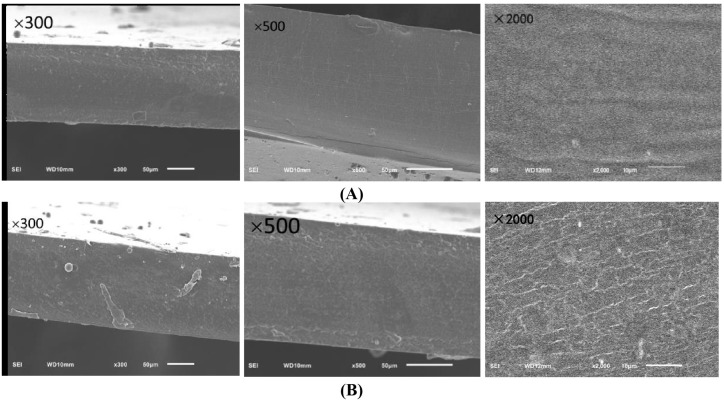
Cross-section and surface microstructure of EG and CG. The grass carp preserved with ZEO-complex polysaccharide gels was the experimental group (EG), and the grass carp preserved with complex polysaccharide gels without ZEO was the control group (CG). (**A**) Cross-section and surface microstructure of EG; (**B**) cross-section and surface microstructure of CG.
